# Blood Pressure and Same-Day Exposure to Air Pollution at School: Associations with Nano-Sized to Coarse PM in Children

**DOI:** 10.1289/ehp.1408121

**Published:** 2015-03-10

**Authors:** Nicky Pieters, Gudrun Koppen, Martine Van Poppel, Sofie De Prins, Bianca Cox, Evi Dons, Vera Nelen, Luc Int Panis, Michelle Plusquin, Greet Schoeters, Tim S. Nawrot

**Affiliations:** 1 Centre for Environmental Sciences, Hasselt University, Diepenbeek, Belgium; 2 Environmental Risk & Health unit, VITO (Flemish Institute of Technological Research), Mol, Belgium; 3 Faculty of Pharmaceutical, Biomedical and Veterinary Sciences, University of Antwerp, Antwerp, Belgium; 4 Environment and Health unit, Provincial Institute of Hygiene, Antwerp, Belgium; 5 Transportation Research Institute, Hasselt University, Hasselt, Belgium; 6 Department of Public Health, Occupational & Environmental Medicine, Leuven University, Leuven, Belgium

## Abstract

**Background:**

Ultrafine particles (UFP) may contribute to the cardiovascular effects of particulate air pollution, partly because of their relatively efficient alveolar deposition.

**Objective:**

In this study, we assessed associations between blood pressure and short-term exposure to air pollution in a population of schoolchildren.

**Methods:**

In 130 children (6–12 years of age), blood pressure was determined during two periods (spring and fall 2011). We used mixed models to study the association between blood pressure and ambient concentrations of particulate matter and ultrafine particles measured in the schools’ playground.

**Results:**

Independent of sex, age, height, and weight of the child, parental education, neighborhood socioeconomic status, fish consumption, heart rate, school, day of the week, season, wind speed, relative humidity, and temperature on the morning of examination, an interquartile range (860 particles/cm^3^) increase in nano-sized UFP fraction (20–30 nm) was associated with a 6.35 mmHg (95% CI: 1.56, 11.14; *p* = 0.01) increase in systolic blood pressure. For the total UFP fraction, systolic blood pressure was 0.79 mmHg (95% CI: 0.07, 1.51; *p* = 0.03) higher, but no effects on systolic blood pressure were found for the nano-sized fractions with a diameter > 100 nm, nor PM_2.5_, PM_coarse_, and PM_10_. Diastolic blood pressure was not associated with any of the studied particulate mass fractions.

**Conclusion:**

Children attending school on days with higher UFP concentrations (diameter < 100 nm) had higher systolic blood pressure. The association was dependent on UFP size, and there was no association with the PM_2.5_ mass concentration.

**Citation:**

Pieters N, Koppen G, Van Poppel M, De Prins S, Cox B, Dons E, Nelen V, Int Panis L, Plusquin M, Schoeters G, Nawrot TS. 2015. Blood pressure and same-day exposure to air pollution at school: associations with nano-sized to coarse PM in children. Environ Health Perspect 123:737–742; http://dx.doi.org/10.1289/ehp.1408121

## Introduction

Air pollution is a complex mixture of solid particles and gases. Different size distribution modes can be identified for airborne particles. Coarse mode particles, with diameters > 2.5 μm, are generally produced by mechanical processes. The formation of ultrafine particles (UFP; particles with a diameter ≤ 100 nm) is often related to combustion or gas-to-particulate conversion. In contrast to PM_2.5_ (particles with a diameter ≤ 2.5 μm), long-range transport is usually not a major source of UFP in urban areas because of the short lifetime of UFP (Sioutas et al. 2005). Relative to larger particles, UFP demonstrate greater cytotoxicity and inflammatory capacity per mass basis. UFP produce a significant inflammatory response in lung cells. The pulmonary inflammatory response induced by UFP may trigger or enhance systemic effects including those of the cardiovascular system (Donaldson et al. 2001; Oberdörster 2001).

Short-term elevation in particulate air pollution has been associated with an increased risk for acute myocardial infarction and stroke (Peters et al. 2001). One important biological mechanism that may contribute to these short-term associations is an air pollution-mediated pro-hypertensive response (Brook et al. 2009). Jacobs et al. (2012) reported that PM concentrations were positively associated with blood pressure measured on the same day in an elderly study population. A French study in pregnant women showed that an interquartile range (IQR) increase in PM_10_ (diameter ≤ 10 μm) was associated with a 1.1% increase in diastolic blood pressure during the first trimester of pregnancy (Hampel et al. 2011). Human exposure studies show that blood pressure increases within only hours of exposure and that blood pressure can remain high until 24 hr postexposure (Cosselman et al. 2012). Chuang et al. (2005) found increases in systolic and diastolic blood pressure in association with 1–3 hr moving averages of submicrometer particles with a size range of 0.02–1 μm in patients with lung function impairment. An IQR increase of 20.8 μg/m^3^ in 24-hr mean outdoor PM_2.5_ was associated with an increase in pulse pressure of 4.0 mmHg in elderly taking anti-hypertensive medication (Jacobs et al. 2012). In a population of adults, present-day levels of PM_10_ and nitrogen dioxide were associated with an increase in blood pressure (Choi et al. 2007). So far, no acute changes in blood pressure in association with PM_10_, PM_2.5_, nitrogen dioxide, or ozone have been found in schoolchildren (Bilenko et al. 2015; Liu et al. 2014; Sughis et al. 2012).

Studies of the effects of air pollution in children have investigated mainly neonatal or infant mortality (Scheers et al. 2011), birth weight (Pedersen et al. 2013), prematurity (Stieb et al. 2012), and respiratory end points such as the incidence of asthma or impaired lung development (Bråbäck and Forsberg 2009). Limited research has evaluated cardiovascular parameters, including peripheral blood pressure, in relation to acute changes in urban pollution in children. In this study we investigated differences in blood pressure of children in association with their exposure at school to a broad span of particles ranging from the nano to the coarse size. Furthermore, to investigate possible underlying mechanisms, an inflammatory marker was measured in exhaled breath condensate (EBC), a relevant matrix to study immediate or short-term responses to air pollution.

## Methods

*Study population*. Children 6–12 years of age were recruited at two primary schools in Antwerp, Belgium, within the framework of the HEAPS (Health Effects of Air Pollution in Antwerp Schools) study (Dons et al. 2014). Children were eligible for inclusion if *a*) they had lived for at least 1 year at their current address, *b*) they were not planning to move residence during the next year, *c*) there was no indoor smoking in their houses, and *d*) their parents were able to complete a questionnaire in Dutch. A subset of 130 children was selected to participate in a study measuring blood pressure. Written informed consent was requested from the parents. Each child was examined twice in periods of about 26 weeks apart: The first sampling period fell within the spring season 2011 (17 May–20 June), whereas the second period fell within fall 2011 (10 November–13 December). Six children dropped out in the second campaign due to change of school or parental refusal. On 3 study days no data on UFP were obtained. This resulted in repeated measurements for 90 children and single measurements for 40 children. Additional information such as the child’s address, age, parental educational status, fish consumption, and travel time from home to school was obtained via questionnaires filled out by the parents the day before the clinical examination. The individual socioeconomic status (SES) was defined as the highest level of education of the mother or the father and was categorized as low (high school not finished), middle (high school finished), and high (higher education or university). In addition, neighborhood SES was assessed using median household income for the year 2011, provided at the statistical sector level by the Belgian National Institute for Statistics (FGOV; Statistics Belgium; http://statbel.fgov.be/en/statistics/figures/). Fish consumption was coded as a categorical variable: never/rarely, one or two times/week, and three or more times/week. Because only a small number of children (4%) had fish consumption of three or more times/week, this category was taken together with one or two times/week. Data on travel time were missing for nine children. The study was approved by the Medical Ethics Committee of Antwerp University.

*Clinical measurements*. The sampling was organized on weekdays between 0900 and 1300 hours in the school. Height and weight were measured while children were not wearing shoes but were fully clothed. Underweight, normal weight, overweight, and obesity were determined based on “Vlaamse groeicurven 2004” (Vrije Universiteit Brussel 2004), which take age and sex into account. After the children had rested for 5 min in a sitting position, a study nurse measured blood pressure by making five to seven consecutive readings using an automated blood pressure instrument (Stabil-O-Graph, Stolberg, Germany) with a pediatric cuff. The guidelines of the European Society of Hypertension were followed for the measurement of blood pressure (O’Brien et al. 2003). The first blood pressure measurement was excluded. The mean of the remaining blood pressure measurements was used for analysis.

*Markers of inflammation*. Interleukin (IL)–1β was measured in EBC. EBC was collected using an RTube™ sampling device (Respiratory Research Inc., Austin, TX, USA). The RTube™ was mounted with an aluminum sleeve that was cooled on dry ice for at least 10 min before collection. Participants were asked to breathe tidally through a mouthpiece connected to the tube during 15 min, yielding approximately 1 mL of EBC. No food was taken 1 hr before collection. After collection, the EBC was immediately divided in aliquots of 250 μL, using 1.5 mL protein LoBind tubes (Eppendorf, Hamburg, Germany). Samples were kept on dry ice and stored at –80°C until further analysis. IL-1β was analyzed using a Meso Scale Discovery Ultra-Sensitive Kit (Meso Scale Discovery, Rockville, MD, USA), which had a detection limit of 50 fg/mL. Samples below the detection limit (21% of all samples) were given a value of 25 fg/mL. Plates were read using a SECTOR® Imager 6000 instrument (Meso Scale Discovery).

*Air pollution monitoring*. Ambient concentrations of UFP, PM_2.5_, and PM_10_ were measured in the playground of each school during the study period; data from 0800 to 1000 hours were used to assess exposure. Air pollution monitoring devices were placed on ground level of the playground. Air was sampled at a height between 1.5 and 2.5 m.

UFP was measured with a Scanning Mobility Sizer (SMPS; model 3080; TSI, Shoreview, MN, USA) and UFP monitor (model 3031; TSI). This latter device allows us to determine the number of particles per size fraction (20–30 nm, 30–50 nm, 50–70 nm, 70–100 nm, 100–200 nm, and > 200 nm). Total UFP fraction was defined as the sum of all separate fractions. The SMPS has a higher size resolution of 64 size bins per decade. Both instruments were compared and the SMPS size distribution was recalculated to the UFP 3,031 size bins.

PM_2.5_ and PM_10_ concentrations were measured with an optical counter (1.108; Grimm, Douglasville, GA, USA), with a sensitivity of 1 particle count/L, a mass resolution of 0.1 μg/L, and a reproducibility of 2%. This device has 15 different size channels for particles with a size between 0.3 and 20 μm. During the monitoring campaign the data were validated to the reference (filter) method using a low-volume sampler (Partisol 2025; Thermo Scientific, Schaumburg, IL, USA), equipped with a PM_2.5_ sampling inlet at a flow rate of 16.7 L/min. PM_coarse_ was defined as the PM_10_ fraction minus the PM_2.5_ fraction (Peng et al. 2008).

UFP data in the playground of their own school were missing for 30 children in the first sampling period and 26 children in the second sampling period. These missing values were imputed using UFP measurements at the playground of the other school (at a distance of 2,800 m). Comparison of simultaneous measurements on both locations showed no significant difference. Temperature, relative humidity, and wind speed from 0800 to 1000 hours were obtained from a local fixed and validated measuring station (42R801) located 2 and 3 km from the examination locations.

*Residential distance to major roads*. The children’s home addresses were geocoded by address; the accuracy was visually inspected using Google Maps and coordinates were manually adapted when they differed from the actual position of the residence. Residential distance to major roads (based on road classification, not intensity) was calculated in ArcGIS 9.3 using the Tele Atlas MultiNet (http://www.spatialinsights.com/catalog/product.aspx?product=95).

*Statistical analysis*. Statistical analyses were conducted using the SAS statistical package, version 9.3 (SAS Institute Inc., Cary, NC, USA) and GraphPad Prism version 5.00 (GraphPad Software, San Diego, CA, USA). Mann–Whitney *U* and Fisher exact tests were used to assess differences between the two schools for continuous and categorical data, respectively. The association between blood pressure and air pollution was examined by treating the pollutants as continuous variables. We used mixed models with random subject effects accounting for repeated measures, assuming a compound symmetry covariance structure. Models were adjusted for the following fixed effects: sex, age, height and weight of the child, parental education, neighborhood SES, fish consumption, heart rate, school, day of the week, season, wind speed, relative humidity, and temperature on the morning of examination. Time-invariant subject characteristics (such as sex and parental education) were included to permit the assumption of a normally distributed random subject intercept. The shape of the association between blood pressure and air pollution and temperature was explored by using natural cubic splines with different numbers of degrees of freedom. Model fit was assessed by using the Akaike information criterion (AIC). The interaction term between season and temperature was explored. For the UFP variables, the single-pollutant analyses described above were repeated with additional adjustment for PM_2.5_ concentrations in the model. *p*-Values ≤ 0.05 were considered statistically significant.

In a series of sensitivity analyses, the model was additionally adjusted for travel time from home to school and for residential distance to major roads. Further, the analyses were repeated excluding imputed UFP data, excluding days with low UFP concentration (total UFP < 5,000 particles/cm^3^) and excluding days with high UFP concentrations (total UFP > 10,000 particles/cm^3^).

The association between IL-1β, a marker of inflammation, and air pollution was examined by mixed models adjusted for the same confounders as before except for heart rate. Estimates [with 95% confidence intervals (CIs)] are presented for IQR increases in pollutant concentrations.

## Results

The study population consisted of 130 children 6–12 years of age (50% girls). Mean (± SD) height was 135.8 ± 10.1 cm and mean (± SD) weight was 30.9 ± 7.9 kg (Table 1). Systolic and diastolic blood pressure averaged 107.1 ± 8.8 and 60.8 ± 7.1 mmHg, respectively. Children of school 1 had higher systolic and diastolic blood pressure, shorter travel time to school, and a slightly lower average household income (Table 1).

**Table 1:** Characteristics of the study population.

Characteristic	All	School 1	School 2
No. of participants	130	63	67
Girls	65 (50)	31 (49)	34 (51)
Age (years)	9.0 ± 1.4	9.0 ± 1.5	8.9 ± 1.4
Height (cm)	135.8 ± 10.1	135.8 ± 10.7	135.8 ± 9.6
Weight (kg)	30.9 ± 7.9	31.8 ± 8.8	30.1 ± 7.0
Body mass index (kg/m^2^)
Underweight	18 (14)	7 (11)	11 (6)
Normal weight	97 (75)	45 (72)	52 (78)
Overweight	11 (8)	9 (14)	2 (3)
Obese	4 (3)	2 (3)	2 (3)
SES indicators
Parental education (individual level)
Low	4 (3)	2 (3)	2 (3)
Middle	28 (22)	15 (24)	13 (19)
High	98 (75)	46 (73)	52 (78)
Household income (aggregated statistical sector; euros)	21347.8 ± 2578.2	20407.3 ± 1600.5	22195.9 ± 2977.3*
Fish consumption
Never/rarely	27 (21)	16 (25)	11 (16)
≥ 1 times a week	103 (79)	47 (75)	56 (84)
Travel time from home to school (min)	11.1 ± 6.8	8.9 ± 4.3	13.3 ± 9.2*
Systolic blood pressure (mmHg)	107.1 ± 8.8	110.4 ± 8.2	104.0 ± 8.1*
Diastolic blood pressure (mmHg)	60.8 ± 7.1	63.1 ± 6.7	58.6 ± 6.8*
Heart rate (beats/min)	83.7 ± 10.3	83.6 ± 10.1	83.8 ± 10.7
Data are given as mean ± SD or *n* (%). *Significant difference between the two schools.			

**Figure 1:**
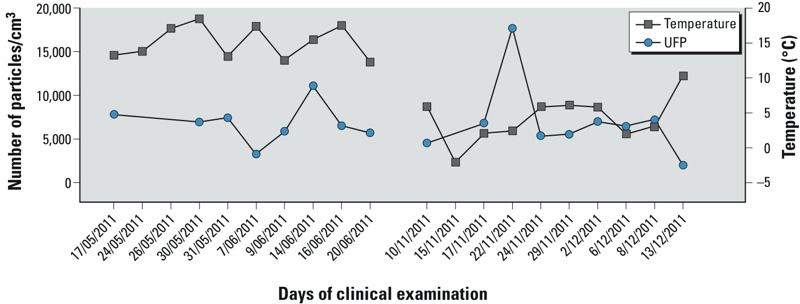
Concentration range of the accumulated UFP fractions (left *y*-axis) and temperature (right *y*-axis) from 0800 to 1000 hours on the day of clinical examination.

**Figure 2:**
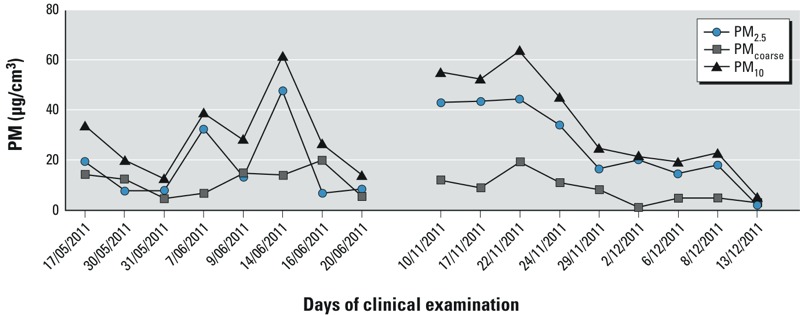
Concentration range for PM_2.5_, PM_coarse_, and PM_10_ from 0800 to 1000 hours on the day of clinical examination.

The mean accumulated concentration for UFP, PM_2.5_, PM_coarse_, and PM_10_ fractions, measured on the different examination days between 0800 and 1000 hours, and the corresponding temperature from 0800 to 1000 hours are given in Figures 1 and 2. The mean relative humidity was 70.3% and 84.0% for the first and second sampling period, respectively. The daily variation in relative humidity is shown in Supplemental Material, Figure S1. The distribution for the different UFP fractions and PM is given in Table 2. Correlations between the different size fractions of UFP and the coarse size fractions are shown in Supplemental Material, Table S1. The largest UFP fractions (100–200 nm and > 200 nm) were significantly correlated with PM_2.5_ and PM_10_; in contrast, the smallest UFP fractions (20–30 nm) were not correlated with PM_2.5_ or PM_10_.

**Table 2:** Exposure characteristics.

UFP/PM fraction	Minimum	25th percentile	75th percentile	Maximum	IQR
20–30 nm (particles/cm^3^)	582	1,018	1,878	2,084	860
30–50 nm (particles/cm^3^)	603	1,637	2,349	4,116	712
50–70 nm (particles/cm^3^)	358	947	1,486	2,886	540
70–100 nm (particles/cm^3^)	203	673	1,031	3,035	358
100–200 nm (particles/cm^3^)	240	666	908	4,601	242
> 200 nm (particles/cm^3^)	33	143	279	1,205	136
Total UFP (particles/cm^3^)	2,020	5,538	7,204	17,701	1,666
PM_2.5_ (μg/m^3^)	2	8	43	53	35
PM_coarse_ (μg/m^3^)	1	5	14	34	10
PM_10_ (μg/m^3^)	5	21	45	64	24

Comparing the fit of the models with a different number of degrees of freedom for air pollution, the association between blood pressure and air pollution showed linearity (data not shown). Temperature, however, was inversely associated with blood pressure at temperatures above approximately 12°C, but not at lower temperatures (data not shown). Models with an interaction term between temperature and season (corresponding to a piecewise linear model with a break point at 12°C) provided the best model fit and were used in further analyses. In spring (temperatures > 12°C) systolic blood pressure for a 1°C increase in temperature was –1.35 mmHg (95% CI: –2.08, –0.63, *p* = 0.0003) lower, whereas in autumn (temperatures ≤ 12°C) the effect of temperature was not significant (–0.09 mmHg; 95% CI: –0.58, 0.40, *p* = 0.72).

Adjusting for sex, age, height and weight of the child, parental education, neighborhood SES, fish consumption, heart rate, school, day of the week, season, wind speed, relative humidity, temperature, and the interaction between season and temperature, systolic blood pressure was significantly associated with UFP fractions up to 100 nm, measured during the morning of clinical examination.

**Figure 3:**
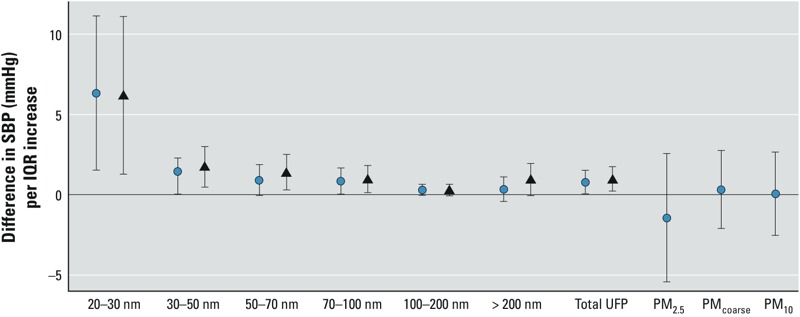
Association between systolic blood pressure (SBP) and different UFP and PM fractions. Estimates (with 95% CI) represent the difference in systolic blood pressure for an IQR (Table 2) increase in the corresponding UFP or PM fraction. Circles show results adjusted for sex, age, height and weight of the child, parental education, neighborhood SES, fish consumption, heart rate, school, day of the week, season, wind speed, relative humidity, temperature on the morning of examination, and the interaction between season and temperature. Triangles are additionally adjusted for PM_2.5_ (only for the different UFP fractions).

Systolic blood pressure was 6.35 mmHg higher (95% CI: 1.56, 11.47, *p* = 0.01) with an IQR increase in the smallest UFP fraction (20–30 nm, IQR = 860/cm^3^; Figure 3). The corresponding associations with UFP fractions of 30–50 nm, 50–70 nm, and 70–100 nm were 1.18 mmHg (95% CI: 0.05, 2.31; *p* = 0.04, IQR = 712/cm^3^), 0.92 mmHg (95% CI: –0.05, 1.89; *p* = 0.07, IQR = 540/cm^3^), and 0.86 mmHg (95% CI: 0.05, 1.68; *p* = 0.04, IQR = 358/cm^3^), respectively, whereas no significant associations effects were estimated for UFP ≥ 100 nm, or for PM_2.5_, PM_coarse_, and PM_10_ (Figure 3). An IQR increase in the total UFP fraction (1,666/cm^3^), was associated with a 0.79-mmHg increase (95% CI: 0.07, 1.51; *p* = 0.03) in systolic blood pressure. When the results were additionally adjusted for PM_2.5_, results were similar (Figure 3). Diastolic blood pressure was not significantly associated with either ultrafine particles or larger particulates (see Supplemental Material, Table S2).

Additional adjustment for travel time to school or for residential distance to major roads gave similar results (Table 3). Associations remained positive and significant when imputed UFP data and days with high UFP concentrations were excluded. (Table 3). However, exclusion of days with low UFP concentrations from the analysis, the associations were no longer significant.

**Table 3:** Sensitivity analysis: estimates in systolic blood pressure (mmHg) for an IQR increase in total UFP concentrations.

Analysis	*n*	β (95%CI)	*p*-Value
Model 1	220	0.79 (0.07,1.51)	0.03
Model 1 + travel time	211	0.78 (0.03, 1.53)	0.05
Model 1 + log distance to major roads	220	0.81 (0.09, 1.53)	0.03
Exclusion of imputed UFP measurements	164	1.27 (0.47, 2.07)	0.004
Exclusion of days with low UFP	182	0.42 (–0.24, 1.07)	0.22
Exclusion of days with high UFP	193	1.96 (0.32, 3.61)	0.02
Data were imputed on 4 days, exclusion of days with low total UFP (< 5,000 particles /cm^3^) on 2 days, exclusion of days with high total UFP (> 10,000 particles/cm^3^) on 2 days.			

Finally, we investigated associations with a marker of lung inflammation. An IQR increase in the smallest UFP fraction was associated with a 24.2% increase in IL-1β (95% CI: 4.83, 47.16, *p* = 0.02), but no association appeared with PM_2.5_ or PM_10_ (Table 4).

**Table 4:** Estimated percent difference in IL-1β (95% CI) per IQR increase in the corresponding UFP/PM fraction.

UFP/PM fraction	IQR	β (95%CI)	*p*-Value
20–30 nm (particles/cm^3^)	860	24.20 (4.83, 47.16)	0.02
30–50 nm (particles/cm^3^)	712	4.27 (–0.56, 9.35)	0.09
50–70 nm (particles/cm^3^)	540	3.79 (–0.30, 8.05)	0.08
70–100 nm (particles/cm^3^)	358	3.28 (0.33, 6.31)	0.03
100–200 nm (particles/cm^3^)	242	1.40 (0.13, 2.68)	0.03
> 200 nm (particles/cm^3^)	136	1.98 (–0.48, 4.49)	0.12
Total UFP (particles/cm^3^)	1,666	2.92 (0.30, 5.61)	0.03
PM_2.5_ (μg/m^3^)	35	–6.28 (–18.54, 7.83)	0.37
PM_coarse_ (μg/m^3^)	10	9.89 (0.17, 20.56)	0.05
PM_10_ (μg/m^3^)	24	–1.33 (–8.91, 6.88)	0.74
Regression coefficients were calculated for an IQR increase in exposure. Models were adjusted for sex, age, height and weight of the child, parental education, neighborhood SES, fish consumption, school, day of the week, season, wind speed, relative humidity, and temperature.			

## Discussion

In this study, children’s systolic blood pressure was positively associated with ambient UFP measured in their school’s playground on the same morning. Associations were statistically significant for particles < 100 nm in diameter, but larger particles, PM_2.5_, and PM_10_ were not significantly associated with blood pressure. To our knowledge, this is the first study of differences in children’s blood pressure in association with different size fractions of PM on the same day. In general, children might be more sensitive to air pollution because of their relatively higher ventilation rate and metabolic turnover, as well as the fact that some of the organ systems including the immune system are still in development (Kim and American Academy of Pediatrics Committee on Environmental Health 2004). Furthermore, their physical behavior, such as greater physical activity, spending more time outdoors, and their closer proximity to traffic exhaust emission sources compared with adults, might add to their vulnerability towards hypertensive effects of airborne particles (Kim and American Academy of Pediatrics Committee on Environmental Health 2004).

Because of their small size, UFP make up only a small fraction of the total PM_2.5_ mass, even though they represent the largest actual number of particles within fine PM. Because UFP have a higher particle number concentration, the surface area is much higher, so they carry large amounts of adsorbed or condensed toxic air pollutants; this results in a different surface chemistry compared with larger particles (Delfino et al. 2005). Therefore, reductions in PM_2.5_ mass may not necessarily reduce the risk of cardiovascular events (Brook et al. 2010). This was also demonstrated in our study. We found that particle size is a determining factor in the effectiveness of particulate pollutants to cause rapid changes in the blood pressure of 6- to 12-year-old children; systolic blood pressure was significantly associated only with exposure to UFP of < 100 nm in diameter; and estimated differences in systolic blood pressure with an IQR increase in exposure decreased with increasing particle size. UFP and PM measurements were performed from 0800 to 1000 hours, and the clinical examination was organized between 0900 and 1300 hours. The mean UFP concentrations for the different fractions were highly correlated between the time frame of 0800–0830 and 0930–1000 hours (correlations > 0.70). Therefore, the reported changes between 0800 and 1000 hours might also reflect the exposure later in the morning.

In contrast with some studies in adults (Wu et al. 2013) and pregnant women (Hampel et al. 2011) or specific patient groups including adults with diabetes (Hoffmann et al. 2012; Jacobs et al. 2012) and the elderly (Chuang et al. 2005), blood pressure was not associated with current PM_2.5_ or PM_10_ concentrations in our study population of children.

In contrast with some previous studies (Choi et al. 2007; Chuang et al. 2005), diastolic blood pressure was not associated with UFP fractions in our study population. It is uncertain why associations with systolic and diastolic blood pressure differ. A possible reason is that systolic and diastolic blood pressures have different regulation pathways and can respond to environmental stimuli in a different way. Although the main physiological role of systolic pressure is to force blood through the arteries during a heartbeat, which is responsive to the sympathetic nervous system and stress stimuli, the role of diastolic blood pressure is to provide perfusion of peripheral tissues during heart relaxation (Paunović et al. 2011).

The clinical significance of particulate-induced increases in blood pressure could be considerable. Childhood blood pressure is an important predictor of hypertension and cardiovascular disease later in life (Hartiala et al. 2012; Juhola et al. 2012; Lurbe and Torró 2010; Lurbe et al. 2005; Raitakari et al. 2003). Although blood pressure is believed to be a complex trait, determined by numerous genetic, biological, behavioral, social, and environmental factors, avoiding or removing potentially irreversible adverse factors as early as possible seems reasonable (Simonetti et al. 2011).

Indeed, repeated particle-induced elevations in blood pressure also lead to repeated increases in arterial wall stress and may result in long-term chronically elevated pressures. Epidemiological evidence exists for a chronic increase in arterial stiffness in children due to traffic-related air pollution, as exemplified by residential traffic-related indicators (Iannuzzi et al. 2010).

Our current epidemiological observations in children are in line with human exposure studies. In a crossover study, where participants were exposed 2 hr to diesel exhaust, increases in systolic blood pressure were reported until 24 hr postexposure. No effects on diastolic blood pressure were reported (Cosselman et al. 2012). Further, a controlled experiment in healthy adults (18–35 years of age) inhaling UFP for 2 hr showed changes in heart rate variability and loss of sympathovagal balance (Samet et al. 2009). Existing evidence suggests that air pollution is able to trigger an acute autonomic imbalance, favoring sympathetic nerve activity causing smooth muscle contraction and thus vasoconstriction (Pieters et al. 2012). In a crossover experiment, systolic blood pressure was significantly lower during a 2 hr walk in Beijing, China, in participants wearing a particulate-filter face mask than in participants who were not protected by a face mask. Wearing the face mask was also associated with increased heart rate variability, which suggests that the rapid increase in blood pressure due to particle inhalation can be mediated through the autonomic nervous system (Langrish et al. 2009). In other controlled studies, ultrafine carbon particles did not change blood pressure or heart rate variability but altered endothelial dysfunction or caused retinal vasoconstriction (Louwies et al. 2013; Routledge et al. 2006; Shah et al. 2008).

Experimental evidence of intratracheally instilled UFP in hamsters showed that UFP can pass from the lungs into the blood circulation within minutes (Nemmar et al. 2001). Due to specific characteristics (high surface area, particle number, metal and organic carbon content) of UFP, they may be transferred directly into the circulation and cause systemic inflammation and peripheral vascular oxidative stress resulting in reductions of nitric oxide, enhancing vasoconstriction and as such change blood pressure. Further, excess production of endothelin-1, a potent vasoconstrictor, after exposure to air pollution, can cause changes in blood pressure (Bouthillier et al. 1998). In animal models, plasma endothelin was up-regulated after exposure to diesel exhaust and concentrated air particles (Vincent et al. 2001). These results were confirmed in an epidemiological setting where patients with metabolic syndrome and healthy volunteers showed an increase in plasma endothelin-1 concentrations 3 hr after diesel exhaust exposure (Peretz et al. 2008).

An association between PM and the development of pulmonary inflammation through proinflammatory cytokine induction has been well documented (Pope and Dockery 2006). IL-1β was measured in EBC, which is useful for detecting early lung inflammation (Garey et al. 2004). In our study population, some UFP fractions were associated with higher IL-1β in EBC. IL-1β can be switched on by activated NF-κB (nuclear factor κB) as an early event of acute inflammatory response, which can subsequently lead to the production of other inflammatory cytokines (Jiménez et al. 2002). Furthermore, the secretion of IL-1β could be considered as an early event in cardiovascular and respiratory illness due to its capacity to induce apoptosis, inhibit myofibroblast differentiation, and repress cell proliferation in rat lung fibroblasts (Zhang and Phan 1999).

Our study has both strengths and limitations. Our study was limited in number of repeated measurements and participants because it was part of a larger biomonitoring program with a fixed design. The UFP concentrations did not differ significantly between the two periods (varied in both periods; see Figure 1); consequently, adaptation toward the blood pressure measurements cannot explain our findings, because variation in exposure was random and independent of the first or second blood pressure reading. To account for diurnal variation in blood pressure, all children were examined at the same moment of the day. To reduce the effect of remaining variability, at least five blood pressure readings were taken after 5 min of rest in the sitting position and the first blood pressure measurement was excluded. Simonetti et al. (2011) reported that parental smoking is an independent risk factor for children’s blood pressure. In this regard, indoor smoking was an exclusion criteria, although this does not account for exposure to passive smoke elsewhere. As reported by Bilenko et al. (2015) and by Liu et al. (2014), noise exposure might be a confounding factor in the association between air pollution and blood pressure. Because we used a repeated-measure design and the child was examined at the same location in both sampling periods and living at the same residential address at the different examinations, noise exposure is unlikely to be a time-varying factor and therefore unlikely to bias our estimates of acute exposure. Additional adjustment for residential proximity to a major road, as a proxy for nighttime noise exposure, did not alter our association between systolic blood pressure and acute UFP exposure (Table 3).

The major strength of the current study is the measurement of the different-sized UFP and PM fractions in school playgrounds to reflect exposure as accurately as possible.

## Conclusion

Children attending school on days with higher ultrafine particulate concentrations (diameter < 100 nm) had higher systolic blood pressure. This association was largely dependent on particle size and was not confounded by the PM_2.5_ mass concentration.

## Supplemental Material

(274 KB) PDF
