# Supplementary material for: Blood Pressure and Same-Day Exposure to Air Pollution at School: Associations with Nano-Sized to Coarse PM in Children
Source: Environ Health Perspect. 2015 Mar 10;123(7):737–42. doi: 10.1289/ehp.1408121 (PMC4492263; doi:10.1289/ehp.1408121)
Supplement: (274 KB) PDF [file ehp.1408121.s001.acco.pdf]

Environ Health Perspect
doi: 10.1289/ehp.1408121

Note to Readers: ***EHP*** **strives to ensure that all journal content is accessible to all readers. However, some figures and Supplemental Material published in *EHP* articles may not conform to 508 standards due to the complexity of the information being presented. If you need assistance accessing journal content, please contact ehp508@niehs.nih.gov. Our staff will work with you to assess and meet your accessibility needs within 3 working days.**

Supplemental Material

Blood Pressure and Same-Day Exposure to Air Pollution at School: Associations with Nano-Sized to Coarse PM in Children

Nicky Pieters, Gudrun Koppen, Martine Van Poppel, Sofie De Prins, Bianca Cox, Evi Dons, Vera Nelen, Luc Int Panis, Michelle Plusquin, Greet Schoeters, and Tim S. Nawrot

Table of Contents

Table S1. Spearman Correlation coefficients between air pollution fractions.

Table S2. Difference in diastolic blood pressure (in mmHg) per IQR increase in the corresponding UFP/PM fraction.

Figure S1. Relative humidity (%) on the day of clinical examination.

Table S1. Spearman Correlation coefficients between air pollution fractions.

	Nano size (#/cm^3)							Coarse size (μg/m^3)		
	**20-30 nm**	**30-50 nm**	**50-70 nm**	**70-100 nm**	**100-200 nm**	**> 200 nm**	**Total UFP**	**$PM_{2.5}$**	**PM_c**	**PM_{10}**
Nano size (#/cm^3)										
20-30 nm	1									
30-50 nm	0.71**	1								
50-70 nm	0.38	0.80***	1							
70-100 nm	0.16	0.61**	0.87***	1						
100-200 nm	-0.11	0.24	0.54*	0.79***	1					
>200 nm	-0.21	0.29	0.52*	0.63**	0.73**	1				
Total UFP	0.62**	0.95***	0.93***	0.80***	0.40	0.40	1			
Coarse size										
$PM_{2.5}$	-0.13	0.32	0.22	0.44	0.53*	0.71**		1		
PM_c	0.11	0.22	0.36	0.36	0.39	0.41		0.23	1	
PM_{10}	-0.15	0.22	0.23	0.4	0.53*	0.69**		0.85***	0.66**	1

Levels of significance were indicated as *$p<0.05$, **$p<0.01$ and ***$p<0.0001$.

Table S2. Difference in diastolic blood pressure (in mmHg) per IQR increase in the corresponding UFP/PM fraction.

UFP/PM Fraction	β (95%CI)	p-value
20-30 nm (#/cm^3)	0.58 (-3.69, 4.86)	0.79
30-50 nm (#/cm^3)	-0.37(-1.41, 0.66)	0.48
50-70 nm (#/cm^3)	-0.26 (-1.14, 0.63)	0.57
70-100 nm (#/cm^3)	0.01 (-0.72, 0.74)	0.98
100-200 nm (#/cm^3)	0.05 (-0.26, 0.36)	0.75
>200 nm (#/cm^3)	-0.27 (-0.95, 0.41)	0.44
Total UFP (#/cm^3)	-0.09 (-0.74, 0.56)	0.80
$PM_{2.5}$ (ug/m^3)	-2.80 (-6.29, 0.69)	0.12
PM_c (ug/m^3)	0.03 (-2.08, 2.15)	0.97
PM_{10} (ug/m^3)	-1.67 (-3.93, 0.58)	0.15

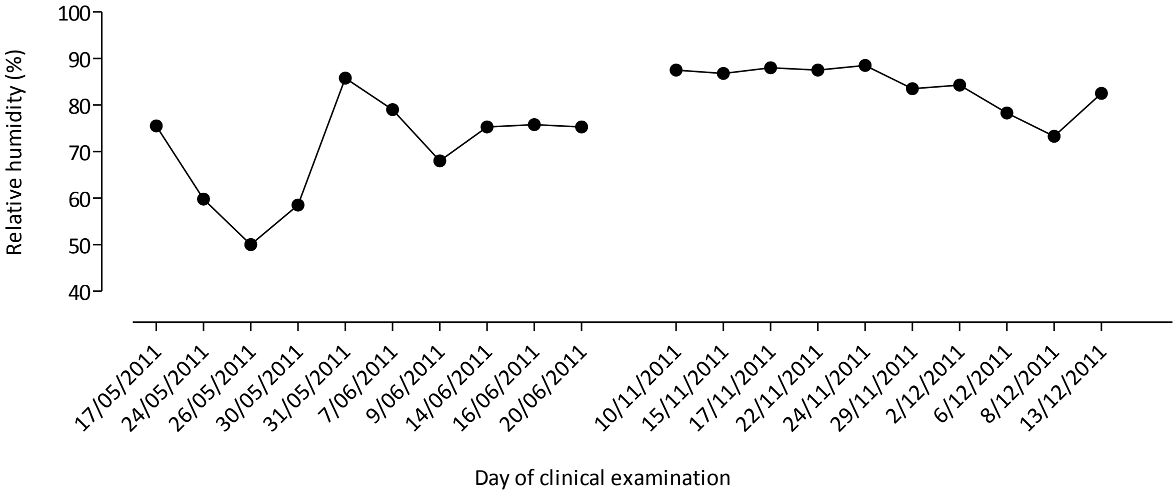

Figure S1. Relative humidity (%) on the day of clinical examination.
